# FoxQ1 Overexpression Influences Poor Prognosis in Non-Small Cell Lung Cancer, Associates with the Phenomenon of EMT

**DOI:** 10.1371/journal.pone.0039937

**Published:** 2012-06-28

**Authors:** Jian Feng, Xuesong Zhang, Huijun Zhu, Xudong Wang, Songshi Ni, Jianfei Huang

**Affiliations:** 1 Department of Respiratory Medicine, Nantong University Affiliated Hospital, Nantong, Jiangsu, China; 2 Department of Pathology, Nantong University Affiliated Hospital, Nantong, Jiangsu, China; 3 Department of Laboratory Medicine, Nantong University Affiliated Hospital, Nantong, Jiangsu, China; Cincinnati Children's Hospital Medical Center, United States of America

## Abstract

**Background:**

We determined the expression of forkhead box Q1 (FoxQ1), E-cadherin (E-cad), Mucin 1 (MUC1), vimentin (VIM) and S100 calcium binding protein A4 (S100A4), all epithelial-mesenchymal transition (EMT) indicator proteins in non-small cell lung cancer (NSCLC) tissue samples. We also investigated the relationship between these five proteins expression and other clinicopathologic factors in NSCLC. Finally, we assessed the potential value of these markers as prognostic indicators of survival in NSCLC's patients.

**Methods:**

Quantitative real-time PCR and immunohistochemistry were used to characterize the expression of the FoxQ1 mRNA and protein in NSCLC. Expression of transcripts and translated products for the other four EMT indicator proteins was assessed by immunohistochemistry in the same clinical NSCLC samples.

**Results:**

FoxQ1 mRNA and protein were up-regulated in NSCLC compared with normal tissues (*P* = 0.015 and *P*<0.001, respectively). Expression of FoxQ1 in adenocarcinoma was higher than in squamous cell carcinoma (*P* = 0.005), and high expression of FoxQ1 correlated with loss of E-cad expression (*P* = 0.012), and anomalous positivity of VIM (*P* = 0.024) and S100A4 (*P* = 0.004). Additional survival analysis showed that high expression of FoxQ1 (*P* = 0.047) and E-cad (*P* = 0.021) were independent prognostic factors.

**Conclusion:**

FoxQ1 maybe plays a specific role in the EMT of NSCLC, and could be used as a prognostic factor for NSCLC.

## Introduction

Lung cancer is the most frequently occurring cancer type, and the leading cause of cancer death globally, with greater mortality than breast, prostate, and colorectal cancer combined [1], [2]. Over the past three decades in China, lung cancer mortality has increased by 465%, with these malignancies becoming the second leading cause of death after liver cancer [3]. Despite great advance in the treatment of cancers in recent years, the prognosis for patients with lung cancer remains poor, with 5-year survival rates less than 15% [4], [5]. Most patients with lung cancer are at an advanced period of the disease at the time of diagnosis, and approximately 85% of these cancers are non-small cell lung cancer (NSCLC) [1], [6].

Many recent studies have noted that the epithelial-mesenchymal transition (EMT) is a critical event in tumour invasion and metastasis in epithelial-derived cancers [7]–[10], including NSCLC [11]–[14]. The awareness of the EMT phenomena dates back as early as 1908. During the 1990s, EMT gained more recognition as a possibly important mechanism in chronic diseases, such as organ fibrosis and cancer [15]. EMT is characterized by down-regulation of epithelial differentiation markers E-cadherin (E-cad) [16]–[20] and Mucin 1 (MUC1) [21], and the up-regulation of mesenchymal markers such as vimentin (VIM) [17]–[20], [22], [23], fibronectin [17], [20], [24] and S100 calcium-binding protein A4 (S100A4) [25]–[28]. Previous studies have described a key role for forkhead box Q1 (FoxQ1) in regulating EMT and aggressiveness in human cancer [29]–[32].

FOXQ1, formerly known as HNF-3/forkhead homolog 1 (HFH1), belongs to a member of the forkhead transcription factor family [32]–[34], which are expressed in different tissues and play important roles in development, metabolism, cancer and aging [30], [34]. As one of the first forkhead genes studied, FOXQ1 has been implicated to repress smooth muscle-specific genes, such as Sm22α and telokin in A10 cells [35]. FOXQ1 has been shown to be a downstream mediator of Hoxa1 in embryonic stem cells [36]. Human FOXQ1, located on chromosome 6p23-25, has been isolated and characterized [33] and plays an essential part in the aetiology of human cancer [31], [32].

**Figure 1:**
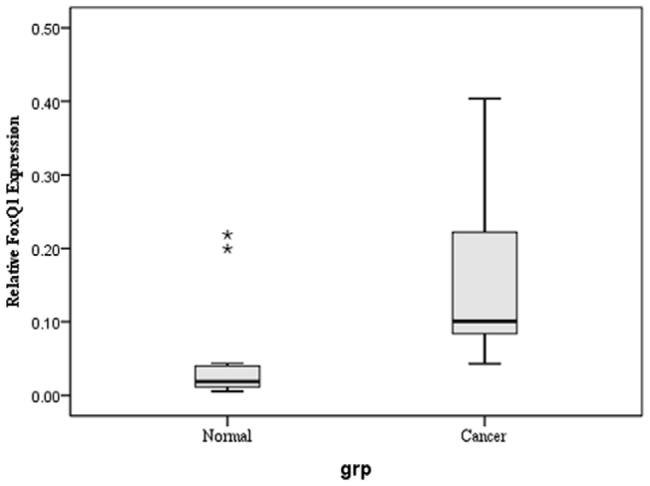
Expression of FoxQ1 mRNA in NSCLC tissues and corresponding non-cancerous tissues. One-step q RT-PCR was performed to confirm the expression of FoxQ1 mRNA in human tissues. Results were normalized to GAPDH mRNA level. The FoxQ1 mRNA level in NSCLC tissues were higher than that in peritumoural tissues with statistical significance using a paired-samples T test. ** *P<*0.05. Bars indicate standard error (S.E.).

**Figure 2:**
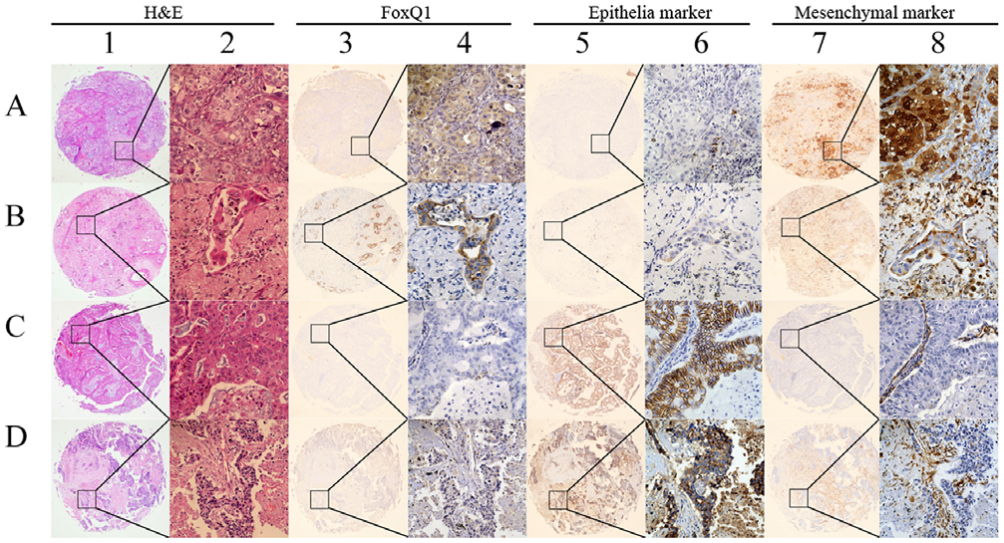
Representative IHC images showing expression of FoxQ1 and EMT-related biomarkers in TMA sections of NSCLC. (A) 1 and 2: lung squamous cell carcinoma tissue pattern with H&E staining; 3 and 4: high expression of FoxQ1; 5 and 6: loss of E-cad expression; 7 and 8: strong VIM-positive staining. (B) 1 and 2: lung adenocarcinoma tissue pattern with H&E staining; 3 and 4: positive staining for FoxQ1; 5 and 6: negative staining for MUC1; 7 and 8: up-regulated expression of S100A4. (C) 1 and 2: lung adenocarcinoma tissue with H&E staining; 3 and 4: negative IHC for FoxQ1; 5 and 6: strong immunological reaction of E-cad; 7 and 8: negative for S100A4. (D) 1 and 2: lung squamous cell carcinoma tissue with H&E staining; 3 and 4: low expression of FoxQ1; 5 and 6: high expression of MUC1; 7 and 8: weak expression of VIM. Original magnification was ×40 for 1, 3, 5 and 7; and ×400 for 2, 4, 6 and 8.

**Table 1:** Correlation of high FoxQ1 expression with clinicopathologic characteristics of NSCLC.

Clinicopathologic characteristics	n	FoxQ1		χ^2^	*P*
		high expression (n)	%		
Gender				0.077	0.782
Male	71	56	78.87		
Female	32	26	81.25		
Age (years)				2.507	0.113
≤60	40	35	87.50		
>60	63	47	74.60		
Tumor diameter (cm)				0.344	0.558
≤3	35	29	82.86		
>3	68	53	77.94		
Histological type				10.709	0.005*
Squamous cell carcinoma	46	30	65.22		
Adenocarcinoma	55	50	90.91		
Others	2	2	100.00		
Differentiation				2.431	0.297
Well	7	6	85.71		
Moderate	66	55	83.33		
Poorly	30	21	70.00		
Lymph node metastasis				1.201	0.548
No regional lymph node metastasis	53	41	77.36		
Metastasis in ipsilateral peribronchial	26	20	76.92		
Metastasis in mediastinal	24	21	87.50		
Stage Grouping with TNM				1.712	0.425
Stage I	50	38	76.00		
Stage II	27	21	77.78		
Stage III and IV	26	23	88.46		

* *P*<0.05.

**Table 2:** Relationship between the expression of FoxQ1 and EMT indicator proteins.

Regular EMT marker expression	FoxQ1 expression		χ^2^	*P*
	Low or none	high (n)		
E-cad			6.308	0.012*
E-cad +	12	23		
E-cad −	9	59		
MUC1			1.396	0.237
MUC1 +	19	65		
MUC1 −	2	17		
VIM			5.073	0.024*
VIM −	20	59		
VIM +	1	23		
S100A4			8.374	0.004*
S100A4 −	12	20		
S100A4 +	9	62		

* *P*<0.05.

**Table 3:** Univariate and multivariate analysis of prognostic factors in NSCLC for 5 year survival.

	Univariate analysis	Multivariate analysis			
	*P*>|z|	HR	*P*>|z|	[95% Conf. Interval]	
FoxQ1 expression					
High vs Low	0.023*	2.091	0.047*	1.009	4.332
Gender					
Male vs Female	0.318				
Age (years)					
≤60 and >60	0.054				
Diameter (cm)					
≤3 vs >3	0.367				
E-cad		2.036	0.021*	1.111	3.730
E-cad+ vs E-cad-	0.002*				
MUC1					
MUC1+ vs MUC1-	0.121				
VIM					
VIM+ vs VIM-	0.666				
S100A4					
S100A4+ vs S100A4-	0.232				
Histological type					
Sq vs Ad	0.980				
Differentiation					
Well vs Moderate and Poorly	0.068				
Lymph node metastasis					
No vs Mip vs Mim	0.174				
Stage Grouping with TNM					
Stage I vs Stage II vs Stage III\IV	0.050				

* *P*<0.05.

Sq, squamous cell carcinoma; Ad, adenocarcinoma; No, no regional lymph node metastasis; Mip, metastasis in ipsilateral peribronchial; Mim, metastasis in mediastinal; HR, Haz. Ratio.

**Figure 3:**
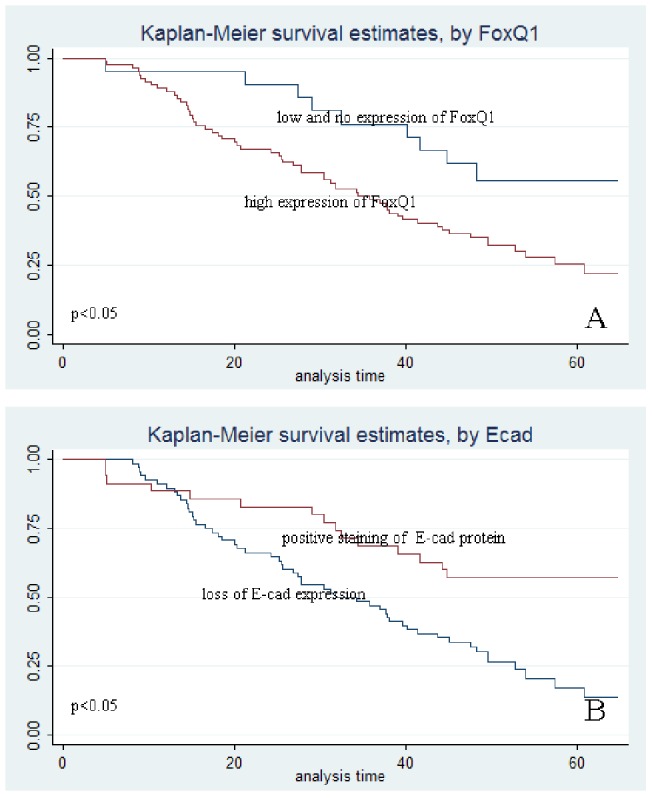
Kaplan-Meier survival curves after surgical therapy in NSCLC. (A) Curves calculated for FoxQ1 expression. High expression in the FoxQ1 group (red line) indicated significantly less survival than low and no expression in the FoxQ1 group (blue line). (B) Curves calculated for E-cad expression. Lifespans of patients with positive E-cad staining are much shorter (red line) than in patients with negative E-cad staining (blue line).

Recent studies have described that FOXQ1 has been found to be overexpressed in colorectal cancer [29], [31] and breast cancer [31], [32], in which patients have poor clinical outcomes [31], [32]. Although the overexpression of FOXQ1 in cancer cell lines confirmed that the gene might play a role in the development of lung cancer [29], [33], the correlation between FOXQ1 expression and EMT factors to determine its clinical significance in NSCLC has not been previously reported.

We analysed expression of the FoxQ1 gene using quantitative reverse transcription polymerase chain reactions (RT-PCRs) in small, freshly frozen NSCLC tissue samples. Expression of the FoxQ1 protein and four common EMT indicator proteins (E-cad, MUC1, VIM and S100A4) was assessed by immunohistochemistry using the same tissue microarray (TMA) sections. Additionally, we investigated the relationship between the expressions of the five genes encoding these proteins and other clinicopathological factors in NSCLC. Finally, we assessed the potential value of these markers as prognostic indicators of survival in patients with NSCLC.

## Methods

### Patients and TMA of NSCLC samples

After a full pathological review according to the 7th Edition of TNM in Lung Cancer [37], a panel of formalin-fixed paraffin-embedded NSCLC tissues with corresponding tumour-adjacent tissues undergoing surgical therapy were obtained from the Affiliated Hospital of Nantong University between January 2005 and December 2006. Clinical data (including gender, age, histological type, grade, stage, tumour size, differentiation, lymph node metastasis status) were obtained from each patient's medical records.

Among the archival material, 103 tissue blocks from NSCLC patients with 5 years' follow-up survival records were available and used for constructing the TMA. A representative area of each tumour was selected and 2.0 mm tissue cores were used to construct a TMA by Shanghai Outdo Biotech (China). The quality of TMA sections was confirmed using haematoxylin-eosin staining (H&E). The average age of the group was 62.5 years (range: 35–81 years). Survival was calculated from the date of surgery until the date of death or last follow-up. Furthermore, a panel of 20 freshly frozen NSCLC tissues and matching peritumour tissues from the same hospital mentioned above were used in this study. Before surgical therapy, none of the patients had received neoadjuvant chemotherapy, radiation therapy or immunotherapy. Ethics approval to perform this study was obtained from the local Human Research Ethics Committee.

### Quantitative RT-PCR (qRT-PCR)

Total RNA was extracted and purified from 40 freshly frozen NSCLC tissue samples, including 20 NSCLC tissues and 20 corresponding non-cancerous tissues. Total RNA extraction, quality control and one-step qRT-PCR were performed as previously reported [38]. FOXQ1-specific oligonucleotide primers (forward, 5′-ACG CTG GCG GAG ATC AAC GAG-3′; reverse, 5′-AGG TTG TGG CGC ACG GAG TT-3′) were designed to yield a 92-bp PCR product. The data were normalized using glyceraldehyde-3-phosphate dehydrogenase (GAPDH) as a reference gene (forward primer, 5′-TCG GAG TCA ACG GAT TTG GTC GT-3′; reverse primer, 5′-TGC CAT GGG TGG AAT CAT ATT GGA-3′).

### Immunohistochemistry (IHC)

IHC was performed as described previously [39]. Deparaffinized sections (4-μm thick) from array blocks were separately stained on an Autostainer Universal Staining System (LabVision, USA) using the following primary antibodies: mouse anti-FOXQ1 (1∶300 dilution; Abcam, UK), mouse anti-E-cad (1∶120; Invitrogen, USA), monoclonal mouse anti-MUC1 (1∶200; Novocastra, UK), monoclonal mouse anti-VIM (1:100; Invitrogen, USA), and polyclonal rabbit anti-S100A4 (1∶100; Newmarker, USA). Secondary antibodies used were: Envision goat anti-mouse HRP (DAKO, USA), Envision goat anti-rabbit HRP (DAKO, USA). The evaluation of immunostaining of these sections was made blind to two trained pathologists who were unaware of the clinical background of the samples.

The percentages of FoxQ1-positive cells were scored and placed into four categories according to staining: 0 for 0%; 1 for 1–33%; 2 for 34–66%; and 3 for 67–100%. The FoxQ1 staining intensities were also scored as: 0, 1, 2, or 3. The sum of the percentages and intensity scores was used as the final FoxQ1 staining score, which we have outlined previously [39] and has been defined as follows: 0–2, low expression; and 3–6, high expression. However, for the positivity of the selected EMT makers (E-cad, MUC1, VIM and S100A4), no detectable or <10% positive staining of tumour cells was deemed as negative, whereas ≥10% positive staining of tumour cells was considered positive [40]. All samples were evaluated at 4 × and 10× magnification.

### Statistical methods

The FoxQ1 mRNA level in freshly frozen NSCLC tissues and corresponding non-cancerous tissues was normalized to GAPDH and analysed using the Wilcoxon signed rank test for Nonparametric Tests. Associations between clinicopathologic variables and FoxQ1 protein expression were examined by χ^2^ tests. The chi-squared were used to confirm the correlation between expression of FoxQ1 and EMT indicator proteins. Survival curves were calculated using the method of Kaplan-Meier and compared using the log-rank test. Factors shown to be of prognostic significance in the univariate models were evaluated using a multivariate Cox regression model. A *P*-value less than 0.05 was considered to be statistically significant. Data were analysed using STATA 9.0 software (Stata Corporation).

## Results

### FoxQ1 mRNA expression in NSCLC and peritumoural tissues

Total RNA was extracted from the freshly frozen NSCLC tissues and subjected to one-step qRT-PCR to investigate FoxQ1 mRNA expression. We also investigated samples from adjacent matched tumour tissues. When normalized to GAPDH, the mean expression levels of FoxQ1 mRNA in NSCLC and corresponding non-cancerous tissue were 0.15±0.02 and 0.04±0.02 (*P* = 0.015), respectively. FoxQ1 expression was 3.75-fold higher on average in the cancer samples than in non-malignant tissues (Fig. 1).

### IHC findings for FoxQ1 and EMT indicator proteins in NSCLC tissues

Typical immunohistochemical staining patterns observed for the five genes encoding the FoxQ1 and the other four indicator EMT proteins in NSCLC are shown in Figure 2. Positive staining for FoxQ1 was mainly localized to tumour cells and pneumocytes in the cytoplasm and plasmalemma at different levels. While positive nuclear staining could be seen, FoxQ1 immunolabelling was not observed in the stroma of these tissues. High FoxQ1 expression was detected in 82/103 (50.49%) of NSCLC tissues and was in 38 (20.39%) of the adjacent matched tumour tissues. The data showed statistical significance using χ^2^ test analysis (χ^2^ = 38.6450, *P*<0.001) and was consistent with FoxQ1 mRNA levels in NSCLCs.

Positive expression of E-cad and MUC1 was localised to the cell membrane, and a combination of the plasmalemma and cytoplasm in NSCLC tumour cells, respectively. Positive immunohistochemical staining for VIM and S100A4 in cancer cells was observed in the cytoplasm, and a combination of the nucleus and cytoplasm. An exception to this was the positive stromal fibroblasts.

### Relationship between expression of FoxQ1 proteins and clinicopathological parameters in NSCLC

The associations between FoxQ1 expression and clinicopathological features of NSCLC are shown in Table 1. FoxQ1 protein expression in adenocarcinoma was higher than in squamous cell carcinoma with statistical significance (χ^2^ = 10.7089, *P* = 0.005) by χ^2^ test analysis. In contrast, no significant associations were seen with patient age, gender, tumour diameter, histological grade of the tumour, lymph node metastasis status, and stage grouping with TNM.

### Correlation between expression of FoxQ1 and the EMT indicator proteins

The relationships between expression of FoxQ1 and the four EMT indicator proteins were calculated and have been outlined in Table 2. It was noted that epithelial protein loss frequencies in the 103 NSCLC tissues were 66.02% for E-cad and 18.45% for MUC1. Abnormal mesenchymal protein expression frequencies in the same samples were 23.30% for VIM and 68.93% for S100A4. The result also showed that high expression of FoxQ1 correlated with a loss of E-cad expression (χ^2^ = 6.308, *P* = 0.012), and anomalous positivity of VIM (χ^2^ = 1.396, *P* = 0.024) and S100A4 (χ^2^ = 8.374, *P* = 0.004) in clinical NSCLC samples.

### Survival analysis

Several known predictive factors of poor outcome in NSCLC were assessed to validate the cohort of patients represented by this TMA (Table 3). High expression of FoxQ1 protein (*P* = 0.023) and low expression of E-cad protein (*P* = 0.002) showed a statistically significant association with five year survival by Cox regression univariate analysis. In addition to these two genetic markers, other NSCLC clinical prognostic factors, such as differentiation of tumour and TNM stage were included in a multivariate Cox regression model. Our data demonstrated that high FoxQ1 expression (*P* = 0.047) and a loss of E-cad expression (*P* = 0.021) were confirmed to be independent prognosticators for low survival of NSCLC.

Survival was plotted using the Kaplan–Meier method. The results identified that the patients with a high FoxQ1 expression or loss of E-cad expression had a significantly shorter survival time, compared to those with low or preserved expression, respectively (Fig. 3).

## Discussion

In this study, using a TMA, we emphasized the prognostic value of FoxQ1 expression in NSCLC. High expression of FoxQ1 was detectable in TMAs of tumour samples and was significantly correlated with decreased overall survival. Furthermore, the results demonstrated that FoxQ1 expression was significantly associated with EMT in a subgroup of patients. Through multivariate analysis, high expression of FoxQ1 and reduced E-cad expression were shown to be independent prognostic biomarkers for poor overall survival. As far as we know, this is the first report of the clinicopathological significance of FoxQ1 expression related to EMT in clinical NSCLC tissue samples.

Recently, accumulating evidence suggests that human FoxQ1 plays a key role in regulating the EMT of breast cancer [31], [32], and aggressiveness in colon cancer [29], [32]. There is considerable proof that presence of the EMT phenomenon indicates short survival in lung cancer [11]–[14]. To identify the relation between FoxQ1 and EMT in lung carcinoma, four frequent indicator biomarkers were investigated in lung cancer TMA using IHC. Interestingly, our results showed that high levels of expression for the FoxQ1 protein correlated with decreased E-cad protein expression, and increases in VIM and S100A4 protein expression.

Some authors have shown that E-cad is linked with metastasis of lung cancer [12]. VIM is not believed to be associated with survival in lung cancer [14], although S100A4 has been correlated with prognosis of lung squamous cell carcinoma [41] in clinical research studies. We also determined the prognostic effect of EMT marker expression by univariate and multivariate analysis. Our results revealed that the only marker associated with outcome was E-cad.

Recent studies have confirmed that FoxQ1 to be a valuable prognostic indicator for poor survival in breast cancer [31], [32]. However, high expression of the FoxQ1 gene was also observed in lung cancer, gastric cancer, and colon cancer cell lines [29]. Thus, our present results corroborate previous findings regarding FoxQ1 expression in NSCLC, especially in lung adenocarcinoma.

Although the exact mechanisms of FoxQ1's tumorigenic effects in NSCLC have not been described fully in our present investigation, the molecular basis for the association between FoxQ1 and EMT are well understood in tumor. The results obtained from our data are in accordance with those presented in the emerging literature, which had declared that the repression of FoxQ1 led to an increase in E-cad expression in human carcinoma [31], [32]. Collectively, the findings in our present study corroborated that FoxQ1 could be potentially used as an EMT marker in NSCLC.

In conclusion, we have shown that FoxQ1 was highly expressed in NSCLC and could be used as a direct prognosticator of a negative outcome. Also, our results supported the fact that FoxQ1 has a functional role with respect to EMT-related genes in NSCLC.
